# Clinic Carpal Tunnel Release Surgery Outcomes and High-Value Care

**DOI:** 10.7759/cureus.42254

**Published:** 2023-07-21

**Authors:** Steven C Kronlage, Mitchell J Lomis, Edward A Whitaker

**Affiliations:** 1 Hand and Upper Extremity Surgery, Andrews Institute for Orthopaedic and Sports Medicine, Gulf Breeze, USA; 2 College of Medicine, Augusta University Medical College of Georgia, Athens, USA; 3 Orthopaedic Surgery, AdventHealth Orlando, Orlando, USA

**Keywords:** field sterilization, clinic, high value care, hand surgery, carpal tunnel release

## Abstract

As healthcare costs continue to rise, the importance of delivering high-value healthcare increases. The volume of carpal tunnel release surgeries performed annually generates a significant cost burden for the healthcare system. The fundamental expenses of carpal tunnel release surgery are facility fees, anesthesia fees, and surgeon fees. Performing open carpal tunnel release surgeries in the clinic utilizing local anesthesia and field sterilization minimizes facility and anesthesia costs. We compared patient outcomes, as measured by infection and revision rates, between hospital-based, ambulatory surgery center-based, and clinic-based carpal tunnel release operations. Three hundred and eighty-eight patients were treated with isolated mini-open carpal tunnel release procedures by three fellowship-trained hand surgeons: 12 patients had hospital-based procedures, 229 had ASC-based procedures, and 147 had clinic-based procedures. All procedures were performed using a mini-open approach. No patients were diagnosed with deep infections post-procedurally, irrespective of venue. Our results show no significant difference in outcomes between venues. Therefore, we conclude that the outcomes of open carpal tunnel release surgeries performed in the clinic were not inferior to carpal tunnel release operations performed at the ambulatory surgery center or the hospital. The cost savings from field sterilization, local anesthesia, and the absence of a facility fee provide an opportunity to expand high-value care.

## Introduction

National implementation of value-based payment models encourages the delivery of high-value care [1]. Under these policies, high-value care can be defined as providing the best care possible while efficiently using resources and achieving optimal results for each patient [2]. Therefore, the expansion of high-value care can be accomplished either by improving clinical outcomes or by achieving existing clinical outcomes while mitigating treatment costs.

Carpal tunnel release (CTR) surgery is a minimally invasive procedure with short operative times. Even without the need for a large operative theater or extensive toolset, the volume of CTR surgeries performed annually places a significant financial burden on the healthcare system [3]. As such, efforts to broaden high-value care have focused on identifying and reducing CTR surgery cost drivers [4].

Fundamentally speaking, three major expenses contribute to the cost of CTR surgery: anesthesia fees, facility fees, and surgeon fees. Wide awake local anesthesia (WALANT), consisting of epinephrine and lidocaine injections, is the least expensive mode of anesthesia as it does not require intraoperative anesthesiologist monitoring. Facility fees have been repeatedly shown to be the least expensive when CTR surgery is performed in a procedure room as opposed to the operating room [4,5]. Even in cases performed without the presence of an anesthesiologist, Leblanc et al. found the cost of CTR surgery in the main operating theater to be more than four times greater than the expense of the procedure room [5]. With recent data showing no difference in clinical outcomes between procedure room and operating room settings after CTR [6], the opportunity to expand high-value care is clear.

Variability between surgical locations and differing sterilization practices undermine the potential facility fee savings and waste reduction that would be gained by shifting CTR surgeries away from the operating room. Cases performed at ambulatory surgery center (ASC) procedure rooms frequently take full sterile precautions, whereas office procedure rooms more often employ field sterilization measures. Additionally, many procedure rooms have anesthesia fees despite the absence of an anesthesiologist. Aiming to optimize resource utilization, our practice began performing open carpal tunnel release (oCTR) in clinic rooms modified for procedures. As the procedures are performed in an office-based setting, no facility fee is charged. Considering the intuitive cost savings, especially over ambulatory surgery center-based procedure rooms, it is important to assess and compare the outcomes oCTR procedures performed in the clinic room with those of other surgical venues.

The objective of the study was to compare outcomes, as measured by superficial infection, deep infection, and revision rates, between patients treated with oCTR in three surgical settings: the clinic procedure room, the ASC procedure room, and the hospital operating room. Additionally, we hope to offer a blueprint for clinic procedure rooms for CTR surgery.

## Materials and methods

Institutional review board (IRB) approval was obtained to perform retrospective outcome and cost analysis of adult (18 years of age) patients that underwent isolated CTR between January 2021 and December 2021 by three fellowship-trained hand surgeons who share the same office practice. Patients were identified using Current Procedural Terminology (CPT) code 64721. A manual chart review was performed to confirm the procedure approach, surgical setting, clinical outcome, and insurance provider. Patients undergoing additional simultaneous procedures were excluded. Demographic data were collected and de-identified. Outcome measures examined were postoperative superficial infection, deep infection, and need for revision. The definition of a superficial infection was a suture abscess or cellulitis of the hand with or without lymphangitis. A deep infection was defined as a wound infection with pus in the depth of the wound that needed incision and drainage or drained spontaneously. The requirement for oral or intravenous antibiotics was recorded.

**Figure 1:**
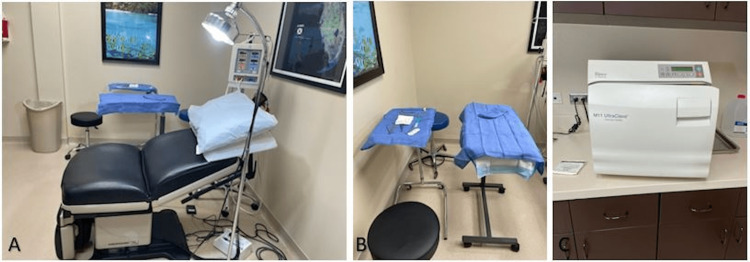
(A) Procedure bed in office, we found a bariatric bed best for our population. Also, one that has an adjustable foot and height makes it easier for elderly patients. (B) We use a Mayo stand and a rolling table draped with blue towels. The arm goes on the rolling table, and the Mayo stand holds the instruments. (C) Office-based autoclave.

All office-based CTR cases in this trial were mini-open procedures (non-endoscopic) performed using field sterility in an office-based setting (Figure 1A). These rooms are used for minor procedures as well as to see patients for routine Evaluation and management during clinical hours. Standard COVID protocol cleaning is performed in all our rooms between patients. No difference exists between cleaning a room for a procedure and cleaning a room for clinical evaluation. There is no defined airflow control. One nurse serves as a circulator and assistant. In this study, field sterility means prepping the hand with alcohol and then with iodine or chlorhexidine. Next, an impervious, sterile towel is used on a Mayo stand to hold the arm (Figure 1B). Sterile blue towels are used as needed. Masks and sterile gloves are used, but the surgeons are not gowned or capped. No prophylactic antibiotics are given unless required by the patient for procedures (e.g., mechanical heart valve, total joint protocol, etc.).

**Figure 2:**
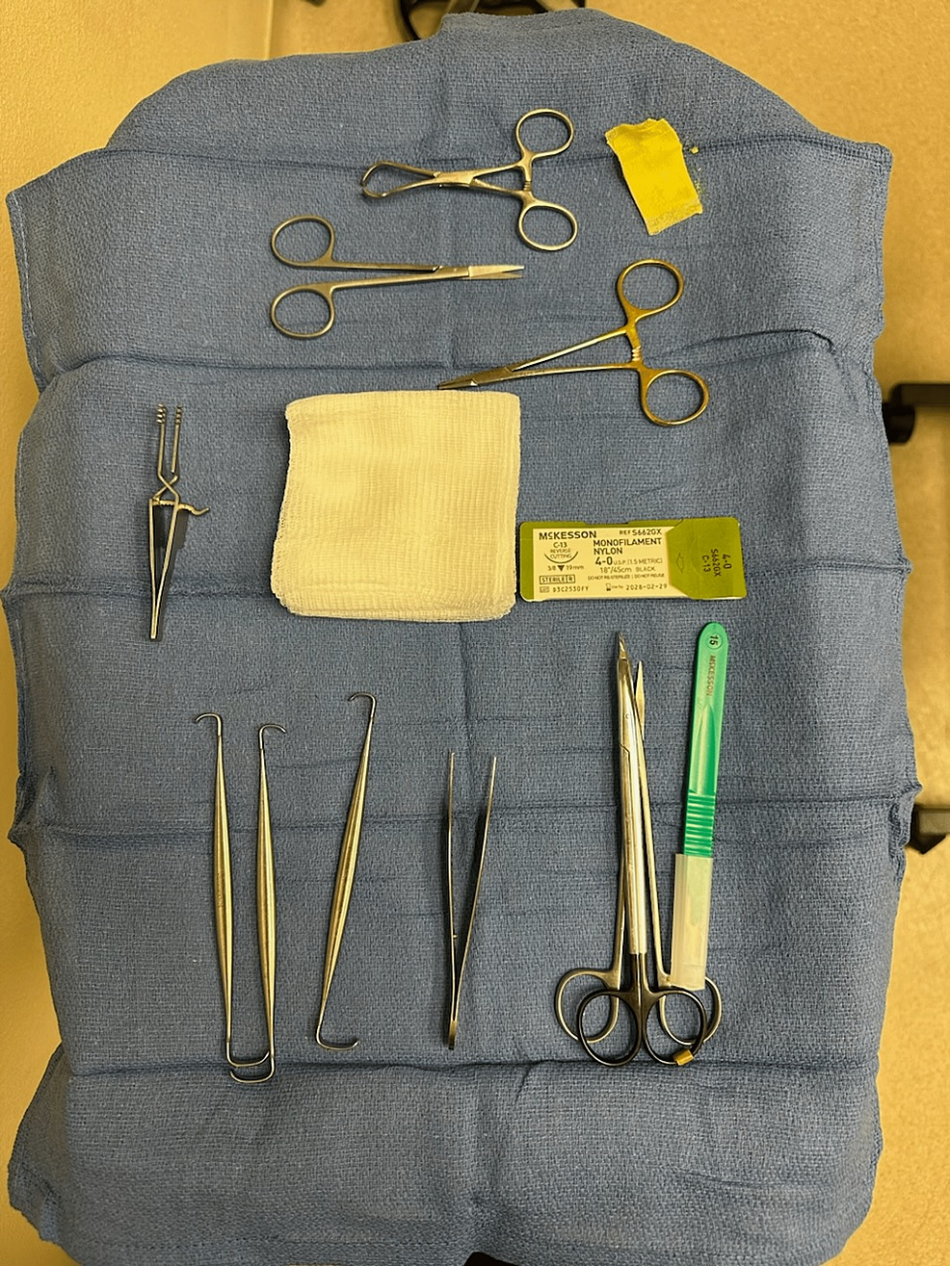
Instruments/materials used for the procedure are shown above: 15-blade Heiss retractor, Ragnell retractor, tenotomy scissors, Adson forceps, suture scissors, towel clip, sterile 4x4 pack, 4-0 nylon suture, xeroform, and Conan wrap

In office-based surgical procedures, there is no sterile processing department (SPD); this must be done in-house with the available staff. Instruments, autoclaves, instrument washing devices, towels, prep, and sutures must be maintained and inventoried (Figure 1C) (Figure 2). A system must be put in place to monitor the autoclave as well as for quality control of instrument cleaning and wrapping. There are commercially available sterile processing consultants that can guide the maintenance of most of this equipment. Most hand surgeons have systems in place for injectables, durable medical equipment (DME), and casting. Office-based surgical procedures can be another way to take care of patients quickly and safely. 

## Results

**Figure 3:**
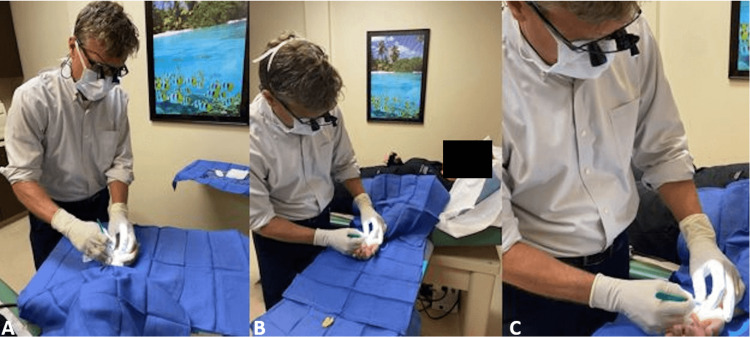
A, B, and C: Examples of field anesthesia. We use a commercially available light on surgical loupes to help with visualization.

Results are shown in Table 1. A total of 388 patients met the inclusion criteria. The average patient age at the time of surgery was 58 years. All patients underwent mini-oCTR. Office-based procedures totaled 147 and were performed with local anesthesia (Figure 3). ASC-based procedures totaled 229 and were performed with general anesthesia, Bier block, or conscious sedation with local anesthetic injection. Hospital-based procedures totaled 12 and were performed with general anesthesia, Bier block, or conscious sedation with a local anesthetic injection. Zero (0) patients in this study were diagnosed with a deep infection. Five (5) ASC patients and three (3) office procedure patients were treated for superficial infections. Revision procedures were required in two (2) office patients (1.36%) and one (1) ASC patient (0.43%). No statistically significant association between superficial infection rates and surgical settings was observed. No bleeding or nerve-related injuries were observed in either group.

**Table 1:** Operative location and postoperative complications.

Variable	Results
Total procedures, n	388
Clinic procedures, n	147
Clinic group superficial infections, n	3
Clinic group deep infections, n	0
Clinic group revisions, n	2
Ambulatory Surgery Center procedures, n	229
Ambulatory Surgery Center group superficial infections, n	5
Ambulatory Surgery Center group deep infections, n	0
Ambulatory Surgery Center group revisions, n	1
Hospital procedures, n	12
Hospital group superficial infections, n	0
Hospital group deep infections, n	0
Hospital group revisions, n	0

## Discussion

This study showed no significant difference in infection or revision rates between patients treated with open carpal tunnel release surgery in the three surgical settings: clinic-based procedure rooms, ambulatory surgery center-based procedure rooms and hospital operating rooms. These findings contribute to the expanding body of literature demonstrating the safety profile of clinic-based procedure room oCTR to be non-inferior to ASC-based procedure room and hospital operating room oCTR operations. 

The possibility of an increased infection rate is a fundamental concern with the use of field sterilization. Leblanc et al. prospectively evaluated 1,504 oCTR procedures performed using field sterility in a minor procedure without antibiotic prophylaxis and reported a superficial infection rate of 0.4% and a deep infection rate of 0% [7]. These rates compare favorably with the data published by Hanssen [8]. We report a superficial infection rate of 2.0% in the clinic room and a deep infection rate of 0.0%. In our study population, this rate was equitable to the 2.1% superficial infection rate of the ambulatory surgery center. 

Long-term patient-reported functional outcome data from Stephens et al. show no difference between procedure room and operating room settings after oCTR [6]. Currently, satisfaction rates have not been compared between these settings for CTR surgery. However, results from Rabinowitz et al. showed significantly higher satisfaction rates among patients treated with trigger finger release in the clinic compared with patients treated in the operating room [9]. Anecdotally speaking, the senior author has had multiple patients transfer from other orthopedic providers to undergo clinic-based CTR surgery instead of ASC or hospital-based CTR surgery. 

The cost advantage of procedure room-based CTR surgery performed with field sterilization has been demonstrated in multiple studies. Comparing overall cost based on facility, Leblanc et al. found the use of the hospital operating room to be almost four times as expensive as the procedure room [5]. Kazmers et al. determined that the total direct cost of performing CTR in the operating room ranged from 6-fold to 29-fold greater than performing CTR surgery in the procedure room [10]. With oCTR facility fee costs increasing by 37% from 2008 to 2016 and out-of-pocket costs to patients rising by 65% over the same period, the decrease in overall healthcare spending and reduction of financial harm to patients are readily apparent [10]. 

With regard to the environment, performing oCTR in the clinic with field sterilization minimizes the procedure’s total carbon footprint. Compared with endoscopic carpal tunnel release (eCTR) performed at the same facility, bivariate analysis by Zhang et al. showed the total carbon footprint of an oCTR to be 46% less than that of an eCTR, with significantly smaller central processing and facility-related carbon footprints [11]. Additionally, disposable material generated with field sterilization is less than 10-fold what is produced in the main operating room [7]. We perform the procedure in the clinic using disposable blue towels instead of drapes. Doing so not only decreases the amount of waste but also substantially decreases costs because the cost of disposing of the waste is significantly diminished.

Despite the identified benefits, matriculation of oCTR surgery out of the operating room and into the office-based procedure room only increased by 1.4% between 2006 and 2017. Over the same period, there has been a 5.4% increase in office-based trigger digit releases, a 10.1% increase in office-based hand or finger mass excision, and a 6.5% increase in office-based hand or finger cyst excision [12]. Randall et al. pointed out potential financial motivations prohibiting the movement of oCTR away from the operating room. With total combined payments for oCTR performed in the operating room averaging 1.2 to 2.4 times more than oCTR performed in the procedure room, surgeons who derive compensation through facility fees from a surgical center or other operational settings may be incentivized to use the operating room more frequently because higher facility fees have a direct impact on surgeon compensation [12]. To circumvent this barrier, policies incentivizing surgeons and health care systems to use the procedure room as a surgical setting may be necessary. Medicare currently incentivizes physicians to perform trigger-digit release procedures in the office setting by reimbursing surgeons 1.78-fold more for office-based operations versus an actual lower reimbursement rate in the ASC or Hospital [13]. Reciprocating this policy with oCTR release may bring transition rates out of the operating room and into the office-based procedure more in line with other minor hand surgeries.

Another advantage not recorded in this study but experienced by the senior author and others is the efficient use of the surgeon’s time. A provider can block time for procedures or see patients in between procedures, making excellent use of clinic time. While doing things faster is not the goal, effective use of time spent operating is important. 

There were limitations in this study. The retrospective design of this study could have caused difficulty in identifying infection rates. To account for this, we examined the medical record for triggers associated with infection, including antibiotic prescriptions, bone or joint cultures, ED/urgent care visits, and consultation with infectious disease specialists. Additionally, we aimed to collect cost data and compare rates between surgical venues. However, we were unable to obtain cost data in the state of Florida for commercial insurance plans. There are different rates for every institution, including ASCs and hospital outpatient departments. These rates are kept secret by the payer and the institution by contract. 

## Conclusions

Moving CTR surgeries away from hospital and ambulatory surgery center-based venues and into an office-based procedure room has the potential to expand high-value healthcare. We showed the safety profile of oCTR surgery performed in a clinic to be non-inferior to its hospital- and ambulatory surgery center-based counterparts. In addition to the repeatedly shown financial benefits, moving CTR surgery to the clinic enhances patient convenience, simplifying the surgical experience akin to dental work. Finally, policy changes mirroring trigger finger reimbursement rates may help increase the number of CTR surgeries performed in the clinic.

## References

[1] Lee VS, Kawamoto K, Hess R (2016). Implementation of a value-driven outcomes program to identify high variability in clinical costs and outcomes and association with reduced cost and improved quality. JAMA.

[2] Razmaria AA (2015). JAMA patient page. High-value care. JAMA.

[3] Hubbard ZS, Law TY, Rosas S, Jernigan SC, Chim H (2018). Economic benefit of carpal tunnel release in the Medicare patient population. Neurosurg Focus.

[4] Brodeur PG, Raducha JE, Patel DD, Cruz AI, Jr. Jr., Gil JA Cost drivers in carpal tunnel release surgery: an analysis of 8,717 patients in New York State. J Hand Surg Am.

[5] Leblanc MR, Lalonde J, Lalonde DH A detailed cost and efficiency analysis of performing carpal tunnel surgery in the main operating room versus the ambulatory setting in Canada. Hand (N.

[6] Stephens AR, Tyser AR, Presson AP A comparison of open carpal tunnel release outcomes between procedure room and operating room settings. J Hand Surg Glob Online.

[7] Leblanc MR, Lalonde DH, Thoma A (2011). Is main operating room sterility really necessary in carpal tunnel surgery? A multicenter prospective study of minor procedure room field sterility surgery. Hand (N Y).

[8] Hanssen AD, Amadio PC, DeSilva SP, Ilstrup DM (1989). Deep postoperative wound infection after carpal tunnel release. J Hand Surg Am.

[9] Rabinowitz J, Kelly T, Peterson A, Angermeier E, Kokko K (2019). In-office wide-awake hand surgery versus traditional surgery in the operating room: a comparison of clinical outcomes and healthcare costs at an academic institution. Current Orthopaedic Practice.

[10] Kazmers NH, Presson AP, Xu Y, Howenstein A, Tyser AR Cost implications of varying the surgical technique, surgical setting, and anesthesia type for carpal tunnel release surgery. J Hand Surg Am.

[11] Zhang D, Dyer GSM, Blazar P, Earp BE The environmental impact of open versus endoscopic carpal tunnel release. J Hand Surg Am.

[12] Randall DJ, Peacock K, Nickel KB, Olsen MA, Kazmers NH (2022). Moving minor hand surgeries out of the operating room and into the office-based procedure room: a population-based trend analysis. J Hand Surg Am.

[13] Medicare: Procedure price lookup: cost. https://www.medicare.gov/procedure-price-lookup/cost/.

